# Role of Cardiolipin in Mitochondrial Signaling Pathways

**DOI:** 10.3389/fcell.2017.00090

**Published:** 2017-09-29

**Authors:** Jan Dudek

**Affiliations:** Department of Cellular Biochemistry, University Medical Center Göttingen, Göttingen, Germany

**Keywords:** Barth-Syndrome, cardiolipin, mitochondria, respiratory chain

## Abstract

The phospholipid cardiolipin (CL) is an essential constituent of mitochondrial membranes and plays a role in many mitochondrial processes, including respiration and energy conversion. Pathological changes in CL amount or species composition can have deleterious consequences for mitochondrial function and trigger the production of reactive oxygen species. Signaling networks monitor mitochondrial function and trigger an adequate cellular response. Here, we summarize the role of CL in cellular signaling pathways and focus on tissues with high-energy demand, like the heart. CL itself was recently identified as a precursor for the formation of lipid mediators. We highlight the concept of CL as a signaling platform. CL is exposed to the outer mitochondrial membrane upon mitochondrial stress and CL domains serve as a binding site in many cellular signaling events. During mitophagy, CL interacts with essential players of mitophagy like Beclin 1 and recruits the autophagic machinery by its interaction with LC3. Apoptotic signaling pathways require CL as a binding platform to recruit apoptotic factors such as tBid, Bax, caspase-8. CL required for the activation of the inflammasome and plays a role in inflammatory signaling. As changes in CL species composition has been observed in many diseases, the signaling pathways described here may play a general role in pathology.

## Introduction

The adult human body hydrolyses 64 kg adenosine triphosphate (ATP) per day. Mitochondria are the primary source of the energy in most tissues and their contribution to energy production is particularly important for tissues with high-energy demand, like neuronal or cardiac tissue. Besides their role in energy conversion, mitochondria have multiple functions in the metabolism, like the citric acid cycle, the urea cycle, the metabolism of amino acids and lipids. Mitochondria also play a role in the biogenesis of heme and iron-sulfur clusters (DiMauro and Schon, 2003). Dysfunctional mitochondria, which cannot provide the required energy particularly affect neuronal tissue and the heart (Wallace, 1999). Cardiomyopathies are frequently associated with defects in respiratory chain subunits and their assembly factors, but also with defects in mitochondrial translation and mtDNA maintenance (Schwarz et al., 2014).

Mitochondria are surrounded by the outer membrane (OM), which allows selective transport of small metabolites and connects mitochondria to other cellular organelles like the endoplasmatic/sarcoplasmatic reticulum (ER/SR), the lysosome and the plasma membrane (Gray, 1963; Elbaz-Alon et al., 2014; Ellenrieder et al., 2017). The inner membrane (IM) separates two compartments, the intermembrane space (IMS) and the matrix. The inner boundary membrane and the cristae structures are distinct functional areas of the IM. The inner boundary membrane is formed by segments of the IM, which approximate the OM in close apposition. The cristae structures are invaginations into the matrix and harbor the respiratory chain. The phospholipid cardiolipin (CL) is a hallmark lipid of mitochondria and almost exclusively found in mitochondrial membranes (Pangborn, 1945). CL is predominantly located in the inner membrane and associated to many mitochondrial functions (see below). Mitochondrial dysfunctions caused by changes in the CL pool are associated to a large number of cardiac diseases (Paradies et al., 2004; Han et al., 2005; Petrosillo et al., 2005; Sparagna et al., 2007; He and Han, 2014; Mulligan et al., 2014). Barth syndrome (BTHS) is an inherited cardiomyopathy, associated with skeletal myopathy, growth retardation and neutropenia and occurs at an estimated frequency of about 1 case per 300,000–400,000 births (Barth et al., 1996; Cantlay et al., 1999; Steward et al., 2010). BTHS is caused by a mutation in the TAZ gene, encoding for Tafazzin, a mitochondrial acyltransferase, involved in the biogenesis of CL (Bione et al., 1996; Dudek and Maack, 2017).

Signaling pathways monitor the physiological state of mitochondria, and trigger a cellular response to various stress conditions. In this review, we describe how alterations in the CL pool cause mitochondrial dysfunction and trigger retrograde signaling pathways. We will discuss the requirement of CL for cellular signaling pathways, such as protein kinase C (PKC) signaling. Upon mitochondrial stress, CL is externalized on the outer mitochondrial membrane forming a binding platform for the specific recruitment of signaling molecules. CL microdomains play a role in autophagy, apoptosis and inflammasome signaling.

## CL is essential for mitochondrial functions

### CL species composition and biosynthesis

Mitochondrial membranes have a characteristic lipid composition, with a high CL content in the IM, where it contributes up to 20% of total lipids (Gebert et al., 2009; Schlame and Greenberg, 2016; Tatsuta and Langer, 2016). The outer mitochondrial membrane also contains CL, where it constitutes up to 3% of the total lipid content (de Kroon et al., 1997). Peroxisomes are the only other organelles, in which a significant amount of CL (2–4% CL of the total phospholipids) was identified (Wriessnegger et al., 2009). CL is composed of a glycerol head group and two phosphatidylglyceride backbones. In total, four fatty acids chains are bound to CL, which differ in length and saturation. In most mammalian tissues, a multitude of different fatty acids bound to CL form a highly diversified CL pool (Maguire et al., 2017). In some tissues, like the mammalian heart, the CL pool consists of a defined species composition with linoleic acid (18:2) being the predominant form for all four acyl chains (Hoch, 1992). It seems that tissue specific metabolism and energy requirements, determine specific fatty acid composition of CL (Paradies et al., 2014). A significant adaption of the CL pool to external stress signaling has been reported in several studies. In yeast, increased total CL levels were found under temperature stress (Luévano-Martínez et al., 2015). A recent study observed increased total levels of CL and increased linoleic acid (18:2) content in CL, in skeletal muscle in response to overload stimuli (Fajardo et al., 2017).

**Figure 1:**
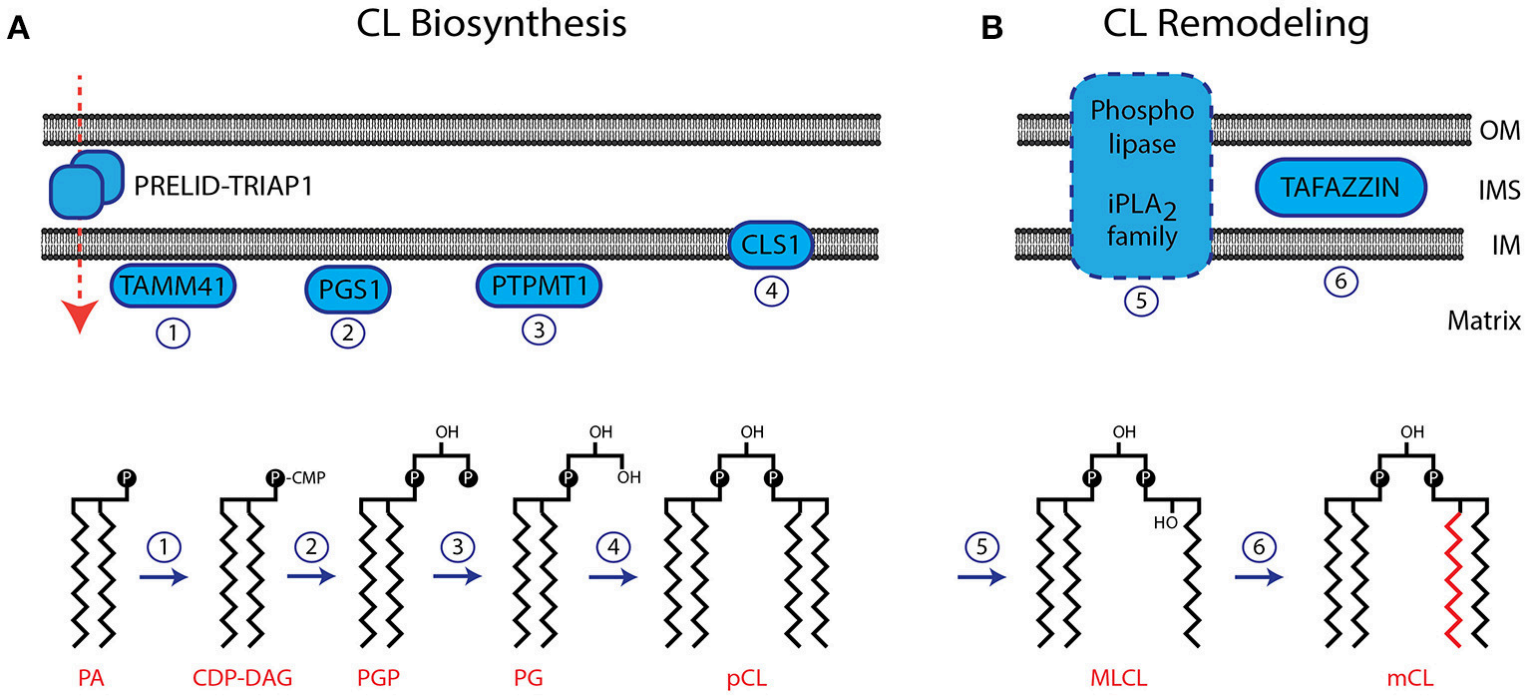
Biosynthesis and remodeling of CL. **(A)** The biosynthesis pathway of CL is localized in the IM. The membrane topology of enzymes involved in CL biosynthesis is indicated. The structures of CL precursor molecules are shown in the lower panel. Numbers of enzymatic reactions correspond with the indicated enzymes in the upper panel. **(B)** CL undergoes remodeling after its initial biosynthesis. The phospholipase, involved in this process has not been identified. Tafazzin is localized in the IMS, associated to both, the IM and OM. OM, outer membrane; IM, inner membrane; IMS, intermembrane space; pCL, premature cardiolipin; mCL, mature cardiolipin.

The IM is the location of the CL biosynthesis and remodeling pathway (Figure 1A). Phosphatidic acid (PA) is imported from the ER and transported across the IMS with the help of the protein complex PRELID-TRIAP1 (Connerth et al., 2012; Potting et al., 2013; Tatsuta and Langer, 2016). After activation of PA by the CDP-DAG synthase TAMM41 (Kutik et al., 2008), the phosphatidylglycerol phosphate synthase (PGS1) catalyzes the committed step by converting CDP-DAG to phosphatidylglycerol phosphate (PGP) (Shen and Dowhan, 1998; Zhong and Greenberg, 2003; He and Greenberg, 2004). Phosphatidylglycerol (PG) is formed by the phosphatase PTPMT1 (Protein-tyrosine phosphatase mitochondrial 1) (Xiao et al., 2011; Zhang et al., 2011). A second molecule of CDP-DAG is used by the Cardiolipin Synthase (CLS1) to form premature cardiolipin (pCL) (Chang et al., 1998; Chen et al., 2006; Lu et al., 2006). After its initial synthesis CL is remodeled by the exchange of fatty acid moieties (Figure 1B). Several phospholipases (iPLA_2_, iPLA_2_γ, iPLA_2_-VIA) have been suggested to deacytylate pCL to form monolysocardiolipin (MLCL) (Mancuso et al., 2007; Malhotra et al., 2009; Yoda et al., 2010; Hsu et al., 2013). The coenzyme A independent acyltransferase Tafazzin then mediates the reacylation to form mature CL (Bissler et al., 2002; Houtkooper et al., 2009a). The gene encoding for Tafazzin is mutated in Barth Syndrome, and the resulting inactivation of this enzyme causes a block in the generation of mature forms of CL (Lu et al., 2016). Despite the remarkable tissue specific composition of CL, isolated Tafazzin was found to have little or no specificity toward fatty acids (Houtkooper et al., 2009b). The specific remodeling of CL arises from the physical properties of the lipid environment and is ensured by the tissue specific availability of substrate fatty acids and the packing conditions of lipids within the membrane (Schlame et al., 2017). Interestingly, Tafazzin is not the only enzyme, capable of CL remodeling. Under pathological conditions, ROS induced upregulation of Acyl-CoA:lysocardiolipin acyltransferase 1 (ALCAT1) causes an aberrant remodeling of CL. ALCAT1 generates CL species with long and polyunsaturated acyl chains such as docosahexaenoic acid (DHA), which are prone to ROS (reactive oxygen species) induced oxidation (Li et al., 2010). ALCAT1 upregulation is associated with hyperthyroid cardiomyopathy and diabetes. The four fatty acid moieties in close proximity make CL susceptible to peroxidation by ROS. As damaged CL is harmful to mitochondria it has been suggested that oxidized CL is rapidly degraded. The CL phospholipase HSD10 has been identified recently to mediate the degradation of oxidized CL (Boynton and Shimkets, 2015).

### The role of CL in mitochondrial protein transport

**Figure 2:**
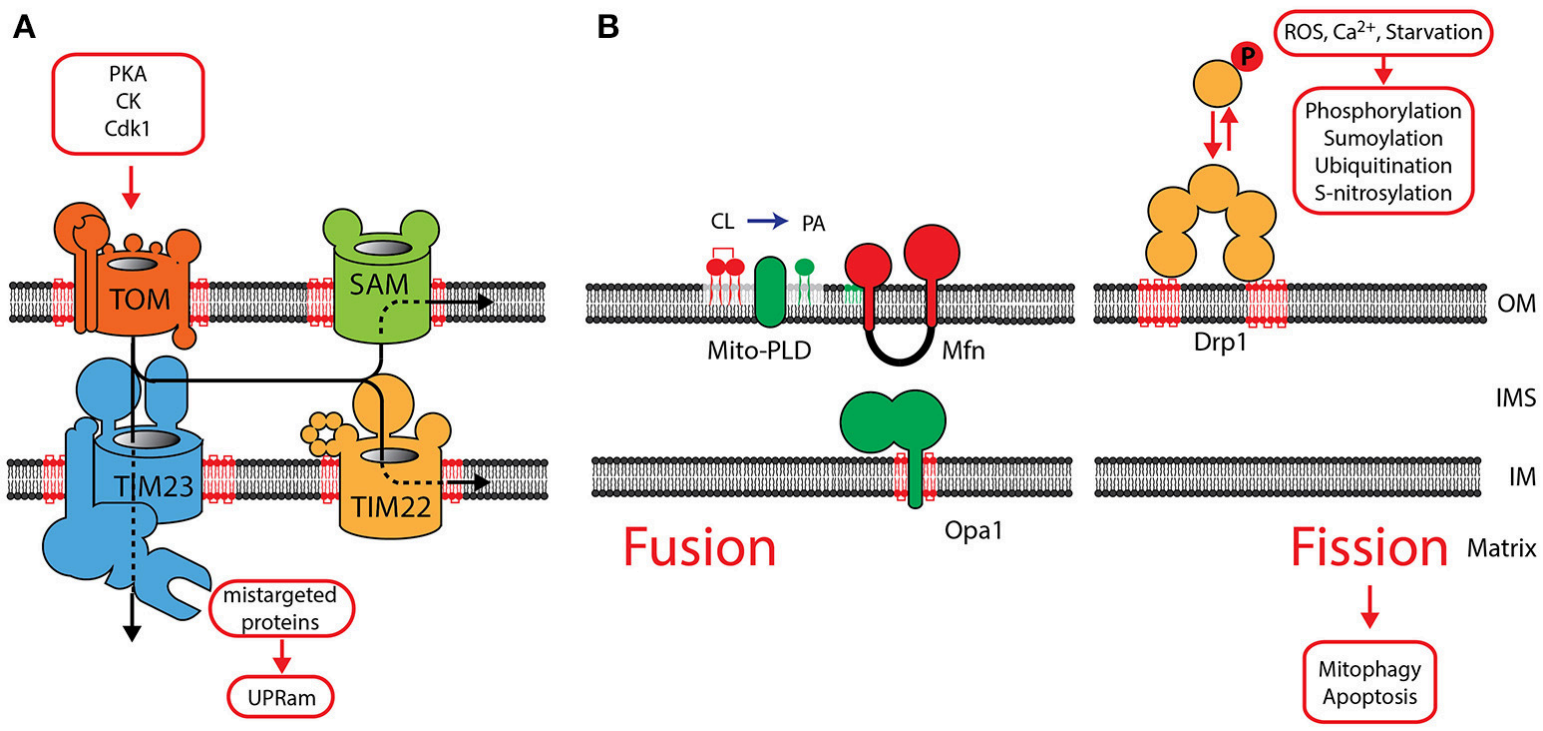
The role of CL in mitochondrial protein translocation and morphology. **(A)** Protein translocases in the OM and IM are dependent on CL. Protein translocation is regulated by phosphorylation of components of the TOM complex. Mistargeted proteins activate the UPRam signaling pathway. **(B)** OM fusion is mediated by MFN1 and MFN2, fusion of the IM requires OPA1. See main text for a detailed description of the role of CL in the function of OPA1. The phospholipase MitoPLD converts CL into phosphatidic acid (PA), which facilitates MFN-mediated fusion. Fission is mediated by recruitment of DRP1 oligomers to the OM, where it interacts with CL. DRP1 oligomerization is intensively regulated by posttranslational modification. OM, outer membrane; IM, inner membrane; IMS, intermembrane space.

Ninety-nine percent of mitochondrial proteins are encoded in the nucleus and transported into mitochondria after their translation in the cytosol. These proteins are transported by specialized translocases across mitochondrial membranes (Figure 2A). The Translocase of Outer Membrane (TOM) serves as a central entry gate for almost all mitochondrial proteins (Neupert and Herrmann, 2007; Dudek et al., 2013b). Protein import is highly regulated by posttranslational modification. Upon a metabolic switch to glycolysis in yeast, Protein Kinase A phosphorylates the TOM receptor subunit Tom70, exerting an inhibitory effect on protein import (Rao et al., 2011; Schmidt et al., 2011). PKA also reduces TOM assembly, whereas Casein kinase and Cyclin dependent kinase (Cdk1) promotes assembly of the TOM complex (Rao et al., 2012; Harbauer et al., 2014). After their import, β-barrel proteins are integrated into the outer membrane by the Sorting and Assembly Machinery (SAM) (Gebert et al., 2009). Inner membrane proteins are transported by the Translocase of inner membrane TIM23 and the associated Presequence Translocase-associated protein import motor (PAM) drives the import of proteins into the matrix (Dudek et al., 2013b). The TIM22 complex in the IM integrates metabolite carriers into the inner membrane. TOM, SAM, TIM22, TIM23 and the associated motor (PAM) require CL for their structural integrity indicating that CL is essential for mitochondrial protein import (Gallas et al., 2006; Tamura et al., 2006; Kutik et al., 2008). AGK has been recently identified a novel structural constituent of the TIM22 complex (Kang et al., 2017; Vukotic et al., 2017). Interestingly, AGK has a catalytic domain, which catalyzes the phosphorylation of DAG to PA. As PA is a precursor for CL, it has been suggested that AGK contributes to CL biosynthesis (Waggoner et al., 2004; Tatsuta and Langer, 2016). Defective mitochondrial import causes protein accumulation in the cytosol, and triggers the unfolded protein response activated by protein mistargeting (UPRam) in yeast (Wrobel et al., 2015). This signaling pathway reduces cytosolic translation and activates the proteasomal degradation pathway. An increase in proteasomal activity in response to a defect in the intermembrane space was also found in mammals suggesting that a similar pathway might also exist in mammals (Papa and Germain, 2011). After import and maturation, cofactors are integrated into mitochondrial proteins, which then acquire their native conformation. CL also plays a role in cofactor integration, as reduced enzymatic activity of Fe-S containing proteins has been reported in CL-deficient yeast (Patil et al., 2013).

### The role of CL in mitochondrial morphology

With four acyl chains bound to one head group, CL adopts a cone-shaped structure and segregates into regions of locally high membrane curvature or even induces membrane bends (Huang et al., 2006). CL is anticipated to localize in the highly bended regions within the cristae structures and preferentially resides in the monolayer facing the matrix side. In addition, the distribution of CL is determined by the lipid scaffolding proteins prohibitin-1 and prohibitin-2 (Merkwirth et al., 2008). Prohibitins belong to the stomatin/prohibitin/flotillin/HflK/C (SPFH) family, which also includes lipid raft-associated proteins. Prohibitin-1 and prohibitin-2 share more than 50% identical amino acids and form large hetero-oligomeric ring-like structures. The somatin like protein 2 (SLP-2) binds to prohibitins and was found to directly interact with cardiolipin (Da Cruz et al., 2008; Osman et al., 2009). Prohibitin complexes are suggested to segregate CL into specialized membrane domains (Merkwirth et al., 2012). A protein with similarities to the family of Hsp40 co-chaperones, DNAJC19, was identified as a prohibitin interaction partner. As DNAJC19 plays a detrimental role for the CL acetylation pattern, it was suggested that the prohibitin/DNAJC19 complex may segregate a specific membrane domain which facilitates Tafazzin mediated CL remodeling (Richter-Dennerlein et al., 2014).

The role of CL in the mitochondrial morphology explains the morphological changes found in cardiolipin-deficient cells. CL deficiency results in an increase in mitochondrial size and absent or disorganized cristae structures (Xu et al., 2006; Acehan et al., 2007, 2009). Mitochondrial morphology is essential for normal mitochondrial function and plays a role in development, aging and apoptosis (Chan, 2006). A large amount of studies implicate changes of mitochondrial morphology in cardiovascular diseases (Brady et al., 2006; Cribbs and Strack, 2007; Williamson et al., 2010).

The complex mitochondrial morphology is determined by several protein structures, in which CL plays a role. The structural organization of the IM and the cristae structures are formed by the mitochondrial contact site and cristae organizing system (MICOS), located at the cristae junctions (Darshi et al., 2011; Gómez and Hagen, 2012). MICOS-deficient mitochondria show a reduction of the number of cristae junctions, resulting in stacks of lamellar cristae. The MICOS complex is formed by nine subunits and interaction partners in the inner membrane, exposing their functional domains into the IMS (Alkhaja et al., 2011; Darshi et al., 2011; Harner et al., 2011; von der Malsburg et al., 2011; Ding et al., 2015; Guarani et al., 2015; Ott et al., 2015; van der Laan et al., 2016). Two proteins MIC26 (APOO) and MIC27 (APOOL) are structurally related to the family of apolipoproteins. MIC27 directly interacts with CL and this interaction was found to be essential for MIC27 assembly into the MICOS complex (Weber et al., 2013; Friedman et al., 2015). The MICOS complex is a central component of a large interaction network which includes several complexes in the IM and OM (Rampelt et al., 2016). MICOS interacts with the protein import and assembly machinery (TOM, SAM in the OM and the IMS resident MIA40), and fusion proteins (OPA1 (see below) and SLC25A46, a mammalian homolog of the yeast fusion adaptor Ugo1) (Abrams et al., 2015; Janer et al., 2016).

Two opposing processes maintain the reticular network of mitochondria: fusion and fission. Fusion (merging of two mitochondria) is mediated by a set of dynamin related GTPases: the mitofusins (MFN1, MFN2) in the OM and OPA1 in the IM (Figure 2B) (de Brito and Scorrano, 2008; Westermann, 2010; Shirihai et al., 2015; Schrepfer and Scorrano, 2016). Their activity is counteracted by fission (segregation of two mitochondria), mediated by DRP1, which also belongs to the family of dynamin related GTPases. Fission and fusion maintains the homogeneity of mitochondria within a cell (Sesaki and Jensen, 1999) and has been shown to be functionally dependent on CL. The fusion protein OPA1 is anchored to the IM by an N-terminal transmembrane domain, exposing the GTPase domain into the IMS. Alternative splicing of OPA1 give rise to two long forms L-OPA1, which are processed by proteases to three short forms S-OPA1 (Ishihara et al., 2006; Anand et al., 2014). A balanced formation of both forms is required for the formation of active dimers and maintaining a reticulated morphology of mitochondria. CL was found to be necessary for the dimerization and induction of the GTPase activity (DeVay et al., 2009; Meglei and McQuibban, 2009). A recent study suggests that the IMS domain of OPA1 contains a CL-binding site. It has been proposed, that after fusion of the OM, this binding domain allows OPA1 to interact with CL in the opposing IM. Therefore, CL- and OPA1-mediated membrane bridging will induce fusion of the IM (Ban et al., 2017). Mitochondrial fission is mediated by DRP1, which is recruited from the cytosol to oligomerize into helical structures. Activation of its GTPase mediates constriction of the outer membrane and induces mitochondrial fission. DRP1 also shows a strong affinity to CL and recent studies indicate that CL binding enhances oligomerization and GTP hydrolysis (Bustillo-Zabalbeitia et al., 2014; Stepanyants et al., 2015). A balanced equilibrium of fusion and fission is essential for maintaining mitochondrial morphology and necessary for mitochondrial inheritance, as recent data in yeast indicate (Böckler et al., 2017). The correct mitochondrial morphology is also a prerequisite for the many interactions of mitochondria with other organelles. Mitofusins act as tethering molecules, which mediate the contact to the ER (Schrepfer and Scorrano, 2016). In cardiac tissue, mitofusins are important for the SR-mitochondrial Ca^2+^ transmission and required for regulating mitochondrial metabolism (Chen et al., 2012).

Based on the functional dependence of the fission and fusion machinery on specific lipids, regulated synthesis and degradation of specific lipids may part of a signaling pathway on mitochondrial membranes. MitoPLD is an OM resident phospholipase, converting cardiolipin (CL) to phosphatidic acid (PA). The generation of PA was found to facilitate mitofusin-dependent fusion of the outer membrane (Choi et al., 2006). PA also recruits the PA phosphatase Lipin 1b, which converts PA to diacylglycerol (DAG). By an unknown mechanism DAG then mediates reduced fusion and increased fission (Huang et al., 2011). Also the phospholipase PA-PLA_1_ was shown to play a role in hydrolyzing PA and counteracting MitoPLD (Baba et al., 2014). The generation of lipid signals might be important to locally define regions of membrane fusion and fission.

### The implication of mitochondrial morphology for cellular signaling

CL plays a fundamental role in forming the shape of mitochondria and mitochondrial morphology is strongly affected in CL-deficient cells. Interestingly, correct mitochondrial morphology is a prerequisite for many cellular signaling pathways, as these pathways are strongly affected in cells with disturbed mitochondrial morphology. Fragmentation of mitochondria in mouse skeletal muscle, induced by the mutation of genes involved in mitochondrial morphology (see above) was reported to cause severe changes insulin signaling, without interference with the contractile functions (Sebastián et al., 2012; Touvier et al., 2015; Wai et al., 2015). Other studies describe skeletal muscle atrophy induced by enforced expression of the fission machinery, which causes AMPK activation and FOXO3 signaling resulting in autophagy activation (Romanello et al., 2010).

Increased fission in apoptotic cells coincides with the release of cytochrome *c* and the execution of apoptosis. Inactivating DRP1 causes a delay in cytochrome *c* release, caspase activation and a block in apoptosis (Frank et al., 2001; Breckenridge et al., 2003; Barsoum et al., 2006). Mitochondrial fission is also necessary for mitophagy in many cell types, as mitochondria are too large for autophagic removal (Arnoult et al., 2005; Twig et al., 2008). Dedicated signaling pathways control mitochondrial morphology during mitophagy. Starvation activates PKA and mediates phosphorylation of DRP1. Phospho-DRP1 is retained in the cytosol resulting in mitochondrial elongation which excludes elongated mitochondria from mitophagy (Cribbs and Strack, 2007; Gomes et al., 2011). DRP1 acts as a central hub in the regulation of mitochondrial morphology and is modified by phosphorylation ubiquitination, sumylation, and S-nitrosylation (Karbowski et al., 2007; Zunino et al., 2007; Cereghetti et al., 2008; Han et al., 2008; Cho et al., 2009; Wu et al., 2011). The stress-induced mitochondrial hyperfusion pathway (SIMH) triggers mitochondrial hyperfusion in response to cellular stress. MFN1 and long isoforms of OPA1 have been found to be required for the SIMH response, which establishes a compensating increase in OXPHOS and ATP levels (Tondera et al., 2009). Interestingly, the CL binding protein Stomatin-like protein 2 (SLP-2) was found crucial for regulating OPA1 processing upon SIMH activation (Christie et al., 2011). SLP-2 was also found to be involved in affect supercomplex stabilization and in segregating cardiolipin into lipid domains the inner membrane (Mitsopoulos et al., 2015).

### Role of CL in the respiratory chain and metabolism

**Figure 3:**
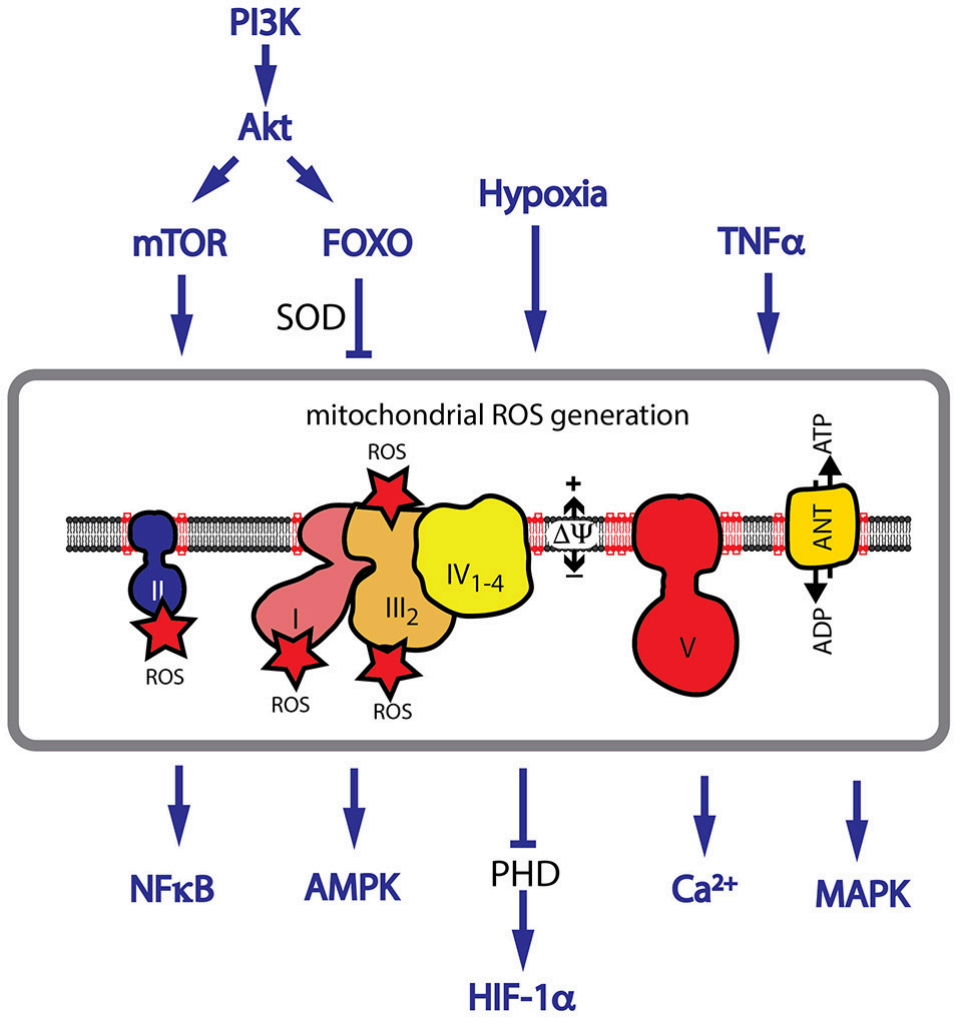
CL deficiency causes an increase in mitochondrial ROS. CL is required for the structural assembly of the mitochondrial respiratory chain. CL deficiency causes a decrease in respiratory performance and an increase in ROS generation. Major pathways implicated in ROS signaling are shown. Hypoxia, TNFα signaling and activation of the PI3K/Akt pathway are associated with an increase in ROS levels. HIF-1α stabilization, and activation of AMPK, NF-κB and many MAPKs are dependent on ROS signaling.

The oxidative metabolism in the cell generates NADH and FADH_2_, which are oxidized by the respiratory chain in the mitochondrial inner membrane. The respiratory chain consists of five complexes (complex I–V) involved in the electron transport to molecular oxygen. The electron transport is coupled with proton export across the IM, generating the membrane potential, which serves as an energy source for the production of ATP by the F_1_F_o_-ATPase. Evidence for CL playing a role in the structural integrity and enzymatic activity exists for all complexes. Structural analysis found specific binding sites for CL in complex I (Fiedorczuk et al., 2016), complex III (Lange et al., 2001; Palsdottir et al., 2003), and complex IV (Itoh et al., 2007). The succinate dehydrogenase (complex II) is strongly reduced in BTHS, indicating a role of CL in the structural integrity of the complex (Dudek et al., 2015). For complex III and complex IV an active role of CL in the proton translocation has been suggested (Eble et al., 1990; Morelli et al., 2013; Duncan et al., 2016). Respiratory chain complexes assemble into large supercomplexes, in which complex I binds a dimer of complex III and several copies of complex IV (Figure 3; Letts et al., 2016; Wu et al., 2016). Supercomplex formation increases the efficiency of the electron translocation and minimizes the risk for the generation of ROS (Schagger and Pfeiffer, 2000). Structural analysis found about 200 CL molecules to be associated with the bovine mitochondrial supercomplex (Pfeiffer et al., 2003; Zhang et al., 2005; Mileykovskaya et al., 2012; Bazan et al., 2013). Analysis of CL-deficient mitochondria indicates a remodeling of supercomplexes with a shift from higher- to lower molecular weight complexes. Therefore, it has been concluded, that CL is required for the structural integrity of supercomplexes (Dudek et al., 2013a, 2015; Huang et al., 2015). CL is also essential for the structure of the family of mitochondrial carrier proteins, which includes the phosphate carrier (PiC), the pyruvate carrier, the tricarboxylate carrier and the carnitine/acylcarnitine translocase (Claypool et al., 2006, 2008; Claypool, 2009).

A large number of phosphorylation sites has been reported for respiratory chain subunits indicating that reversible phosphorylation plays a prominent role in regulation of respiration (Covian and Balaban, 2012). Other covalent modifications of respiratory chain subunits include the modification by lysine acylation and succinylation, which is reversed by the family of mitochondria-localized NAD^+^-dependent deacetylases SIRT3, SIRT4, and SIRT5. SIRT3 was shown to regulate subunits in all five respiratory chain complexes upon dietary challenges such as calorie restriction (Bao et al., 2010; Cimen et al., 2010; Kim et al., 2010). SIRT5 regulates the respiratory chain by desuccinylation (Zhang et al., 2017). A specific binding of an N-terminal amphipathic helix in SIRT5 to CL was recently found to be essential for its activity (Zhang et al., 2017).

## Signaling pathways affected by CL

### Mitochondria play a central role in ROS signaling

The structural remodeling of the respiratory chain in BTHS is associated with the aberrant transfer of electrons onto O_2_, forming superoxide (^.^${\text{O}}_{2}^{-}$) and other forms of ROS (Dudek et al., 2015). When ROS defense mechanisms are overwhelmed, ROS directly reacts with lipids, proteins and DNA (Davies, 1995). ROS also targets proteins of the respiratory chain, affecting its structure and initiates a vicious cycle by further generating more ROS (Nickel et al., 2014, 2015). ROS-induced cellular damage is associated with many forms of cardiac disease in aging (Gómez and Hagen, 2012), ischemia/reperfusion (Ostrander et al., 2001) and heart failure (Saini-Chohan et al., 2009).

A role for ROS as a second messenger in cellular signaling pathways has also been described. ROS activates signaling proteins by changing their redox status. The most susceptible molecular targets of ROS are cysteine and methionine residues. Oxidation of cysteine causes intra- or intermolecular disulfide formation or nitrosylation and glutathiolation. Other posttranslational redox modifications include the hydroxylation of proline and arginine and the nitration of aromatic amino acids. ROS have been integrated as second messengers in many fundamental signaling pathways, modulating the cellular response. Activation of many pathways including the PI3K/Akt pathway and TNFα signaling are associated with an increase in ROS levels (Nogueira et al., 2008). The balance between ROS generating and ROS detoxifying mechanisms are crucial for the activation of downstream pathways. Hypoxia is associated with an increase in ROS levels in many cell types and ROS was identified as an essential contributor to the stabilization of the transcription factor HIF-1α (Diebold and Chandel, 2016). Other transcription factors, which are reported to be activated by ROS include NF-κB, FOXO3A, p53, and PGC1α (Gloire and Piette, 2009; Chae et al., 2013; Marinho et al., 2014). Upstream signaling components are also regulated by ROS, like the Protein kinase C (PKC), which is directly activated by oxidation of cysteine residues (Gopalakrishna et al., 1997). In cardiac hypertrophy AMPK and many MAPKs including p38, JNK, apoptosis-signaling kinase 1 (ASK-1) and ERK1/2 (Giordano, 2005) are activated by ROS. Mitochondria have the ability to significantly amplify a local increase in ROS levels. In a process called ROS-induced ROS release the opening of the mitochondrial permeability transition pore (MPT) and the inner membrane anion channel (IMAC) induces a ROS burst, which not only induces redox-sensitive signaling pathways but also triggers a similar ROS burst in neighboring mitochondria (Zorov et al., 2014).

### Role of CL in MCU and Ca^2+^ signaling

Mitochondrial morphology is critical for many signaling pathways. During excitation-contraction coupling, Ca^2+^ emitted from the sarcoplasmic reticulum is transmitted into the mitochondrion. Ryanodine receptors (RyR) in the sarcoplasmic reticulum are in close proximity to mitochondria and the mitochondrial calcium uniporter (MCU) in the inner membrane. The formation of this functional microdomain has been considered to be essential for the efficient transmission of Ca^2+^ into mitochondria. Transmission of Ca^2+^ into mitochondria was absent in fragmented mitochondria (Szabadkai et al., 2004).

Ca^2+^ plays a prominent role in the regulation of mitochondrial metabolism allowing adaptation to increased energy demands during increased cardiac workload (Dorn and Maack, 2013). Ca^2+^-mediated activation of the pyruvate dehydrogenase, the isocitrate dehydrogenase and the α-ketogrutarate dehydrogenase triggers a significant increase in the metabolic flux in the Krebs cycle. At the same time respiration is increased by activation of complex III, IV and V. The Ca^2+^ conducting pore of the mitochondrial Ca^2+^ uniporter (MCU) is formed by MCU and EMRE (Baughman et al., 2011; De Stefani et al., 2011; Sancak et al., 2013; Oxenoid et al., 2016). A novel form of regulation has been discovered recently with the finding that oxidative stress causes S-glutathionylation and persistent activity of MCU (Dong et al., 2017). MCUb was described as a dominant negative component of the Ca^2+^ channel (Raffaello et al., 2013). Regulation of MCU as a calcium induced calcium channel is mediated the Ca^2+^ sensitive subunits MICU1 and MICU2 (Petrungaro et al., 2015). This regulatory unit is bound to MCU complex via its interaction with EMRE (Tsai et al., 2016). Recently, MICU1 and MICU2 was found to interact with CL, indicating that the binding to CL also contributes to the membrane association (Kamer et al., 2017). Studies in cells, deficient of the regulatory subunits MICU1 and MICU2 revealed a deregulated calcium uptake into mitochondria (Patron et al., 2014). If deregulated Calcium uptake plays a role in BTHS has not been investigated.

### Mitochondrial protein kinase C signaling depends on CL

Human cells have fifteen isoforms of the Protein kinase C (PKC). Overstimulation of some isoforms are associated with hypertrophy in cardiomyocytes, other forms are associated with a cardioprotective function (Mellor and Parker, 1998). One of the first data of a direct role of CL in PKC signaling was generated in yeast. CL-deficient yeast was found to have a defect in the integrity of the cell wall. Cell wall biogenesis and intracellular turgor pressure are regulated by the PKC-Slt2 and HOG pathway, respectively. CL deficiency caused a defect in the phosphorylation and activation of the MAPK Slt2, the downstream effector of PKC (Zhong et al., 2005, 2007; Zhou et al., 2009). Overexpression of individual genes involved in the PKC-Slt2 pathway rescued the mutant phenotype. As deletion of components of the HOG pathway also rescued the phenotype, it was concluded, that the balanced homeostasis of both pathways is affected in CL-deficient yeast (Patil and Greenberg, 2013). The PKC-Slt2 and the HOG pathway in yeast are also involved in the induction of mitophagy. Recent data indicates that defective activation of both pathways contribute to the described mitophagy defect in CL-deficient yeast (Shen et al., 2017). It is presently unclear, if a direct binding of CL may regulate PKC and Slt2 in yeast or loss of CL has an indirect effect on the components of the Slt2 pathway.

In the heart, several targets for PKC have been identified including the myofilament proteins troponin, cMyBP-C, titin, and the cytoskeletal protein desmin (Hidalgo et al., 2009; Kooij et al., 2010). Although most members of the PKC family are cytosolic proteins, the two diacyglycerol (DAG) sensitive but Ca^2+^ insensitive PKCε and PKCδ localize to the inner mitochondrial membrane (Budas et al., 2010; Yang et al., 2012). These proteins were found to interact with the cytosolic chaperone HSP90 and the mitochondrial import receptors TOM20 and TOM70. This led to the model of a regulated import into mitochondria upon mitochondrial stress, such as ischemia and reperfusion. Activation of mammalian PKCε was found to be dependent on CL (Konno et al., 1989; Shen et al., 2017). In mitochondria PKC was suggested to regulate mitochondrial proteins, involved in glycolysis, TCA cycle, β-oxidation, and ion transport and it physically interacts with regulatory subunits of the respiratory chain (Ping et al., 2001; Baines et al., 2003). Interestingly, PKCδ was shown to phosphorylate and activate the phospholipid scramblase 3 (PLS3), which is an important mediator of CL externalization (see below), revealing an interesting interdependence of CL and PKC (He et al., 2007). By supporting complex IV activity and preventing pathological ROS release, PKCε was suggested to have a cardioprotective effect (Guo et al., 2007; Yu et al., 2008).

### CL is precursor for lipid mediators released under stress conditions

Lipid mediators such as prostaglandins, thromboxanes, leukotrienes play a role in many pathological processes such as inflammation, fewer, allergy and other immune responses. These signaling molecules are derived from phosphatidylserine (PS) by PLA_2_-mediated (Ca^2+^ dependent phospholipases A_2_) release of polyunsaturated fatty acids, which are subsequently oxidized by cyclooxygenases (COX) and lipoxygenases (LOX). Mitochondrial CL was recently found to be the origin of a new class of mitochondrial mediators. In a very similar process, cytochrome *c*-mediated oxidation of CL has been proposed to play a role in the production of specific lipid mediators under stress conditions. Oxidized CL was suggested to be a precursor for iPLA2γ-mediated release of oxidized polyunsaturated fatty acids and the accumulation was inhibited by specific inhibitors of iPLA2γ (Tyurina et al., 2014; Liu et al., 2017). Typical mediators, released by this process are 9-Hydroxyoctadecadienoic acid (9-HODE) and 13-HODE. A functional role for lipid mediators in mitochondria is not known, yet. Targets for 9-HODE and 13-HODE have been found predominantly in the cytosol where they were proposed to activate G2A, a G protein-coupled receptor and TRPV1 (Transient receptor potential vanilloid 1), which is expressed in the peripheral and central nervous systems (Patwardhan et al., 2009; Ogawa et al., 2010).

## Externalized CL forms binding sites for signaling molecules

### Mechanisms of CL translocation into the OM

The specific exposure of phosphatidylserine (PS) on the plasma membrane serves as a well-studied “death signal” in apoptosis. In healthy cells, PS is maintained in an asymmetrical distribution on the cytosolic site of the plasma membrane. The activation of a scramblase upon apoptosis externalizes PS on the outer surface forming a recognition signal for macrophages. The unique biochemical properties of CL have fostered the hypothesis that CL enriched membrane sites might also serve as a binding site for specific protein complexes. Two negative charges allow the specific recruitment of protein interaction partners. CL, which is translocated from the IM and exposed on the OM under stress conditions, will serve as a binding site for signaling molecules and establishes a signaling hub for mitophagy and apoptosis.

**Figure 4:**
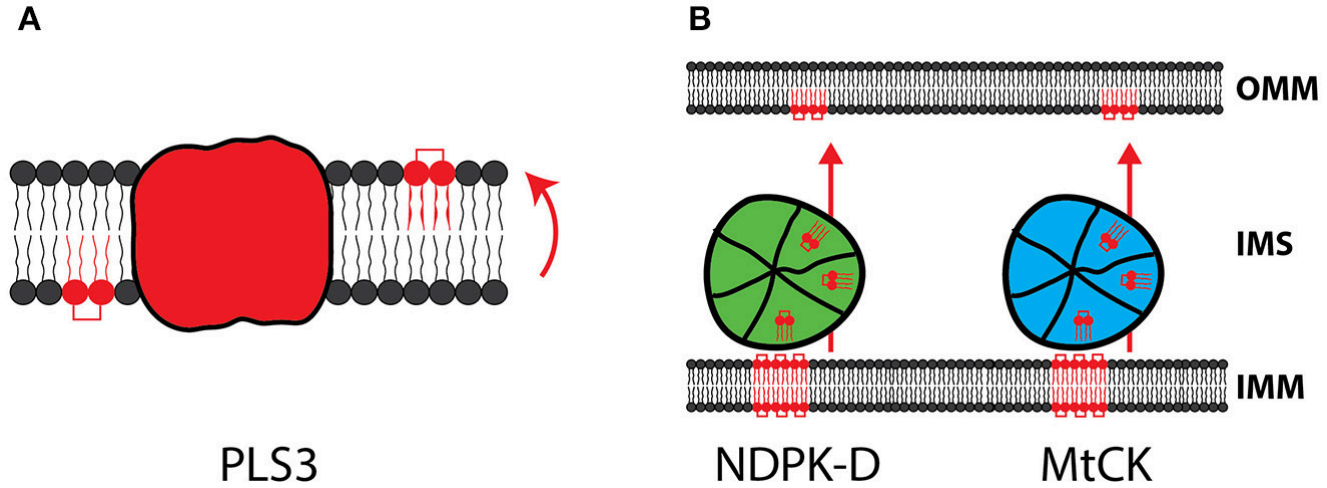
CL trafficking from IM to OM. **(A)** The mitochondrial scramblase PLS3 allows the translocation of CL from the inner to the outer leaflet of the membrane. **(B)** NDPK-D and MtCK form large oligomeric complexes in the IMS and are capable to transport CL from the IM to the OM.

In the inner mitochondrial membrane CL shows an asymmetrical distribution with a majority of CL facing the matrix. Phospholipid scramblases (PLS) are a small family of phospholipid translocators, which mediate the translocation of phospholipids between the two monolayers of a lipid bilayer (Figure 4A). Mitochondrial phospholipid scramblase-3 (PLS-3) is involved in the redistribution of CL within the membrane, which is considered a necessary step for translocation of CL to the outer membrane. Remarkably, inactivation of PLS-3 causes increased resistance to apoptosis, highlighting the importance of CL translocation for cellular signaling (Liu et al., 2003; Van et al., 2007).

Two IMS proteins, with an established role in energy metabolism, were recently found to have a novel function in the transport of CL into the outer membrane. The Mitochondrial creatine kinase (MtCK) provides the cellular energy buffer phosphocreatine by ATP dependent phosphorylation of creatine. The nucleoside diphosphate kinase (NDPK-D/NM23-H4) converts nucleoside diphosphates to triphosphates by transferring the terminal phosphate group. Both enzymes were found to form large oligomeric complexes in the IMS, exposing specific binding sites for CL (Figure 4B). By binding to CL, these proteins were shown to mediate CL transfer between the inner and outer membrane (Lacombe et al., 2009; Schlattner et al., 2013).

### CL externalization in mitophagy

Mitophagy is the selective degradation of dysfunctional mitochondria by autophagy. Mitophagy plays an essential role during cardiac development and maintains cardiac function in the adult heart (Billia et al., 2011; Hoshino et al., 2013; Kubli et al., 2013; Gong et al., 2015). During mitophagy, damaged mitochondria are enclosed by a double membrane structure, the phagophore, which forms the autophagosome and fuses with the lysosome to target its content for degradation (Youle and van der Bliek, 2012). Due to their size, mitochondria have to be separated from their reticular network in order to be incorporated into the autophagosomes. Mitochondrial fission has been shown to be a prerequisite for mitophagy in many cell types. Inhibition of mitochondrial fission decreases the level of mitophagy (Thomas and Jacobson, 2012). The two CL dependent proteins DRP1 and OPA1 activity mediate the balanced fusion and fission of mitochondria (see above). Interestingly, another CL binding protein is involved in mitochondrial fission during mitophagy. The mitochondrial human immunity-related GTPase, IRGM, was shown to play an essential role in mitophagy induced fission and translocates from the cytosol to mitochondria, upon mitophagy induction, where it binds to CL (Singh et al., 2010).

The concept of CL externalization forming an essential platform for mitophagy was emerging from the observation that CL translocates to the OM upon CCCP treatment, a well-studied inducer of mitophagy in mammalian cells. Both the content and species composition of CL in the OM were increased upon mitophagy induction (Chu et al., 2013). Externalized CL plays a central role in mitophagy. Phagophore formation is promoted by activation the class III phosphatidylinositol 3-kinase Vps34, which generates phosphatidylinositol-3-phosphate PtdIns(3)P, required for the recruitment of further effectors (Kaur and Debnath, 2015). Vps34 forms a complex with Beclin 1, Vps15, and Ambra1. Beclin 1 is a central regulator of this complex and multiple proteins interact with Beclin 1 to induce or inhibit autophagy (Kroemer et al., 2010). Beclin 1 has been shown to directly interact with CL on the OM, indicating a direct involvement of CL in mitophagy (Huang et al., 2012).

**Figure 5:**
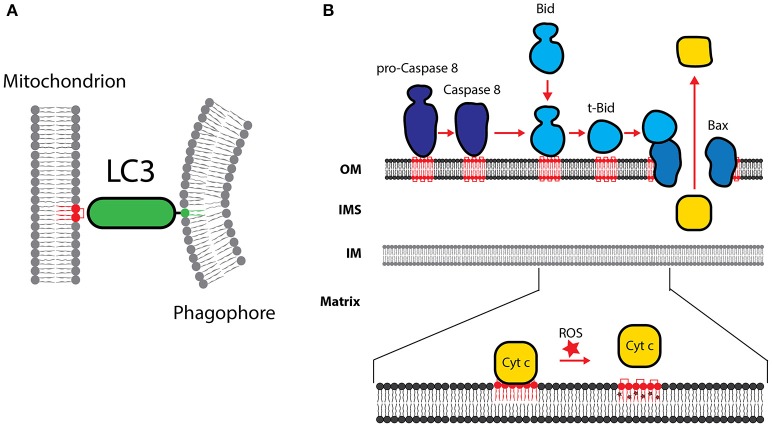
Cardiolipin in apoptosis and mitophagy. **(A)** CL is necessary for the recruitment of LC3, which mediates binding of the phagophore membrane. **(B)** The role of CL in the processing of procaspase-8 is shown. Caspase-8-mediated processing of Bid to form t-Bid is stimulated by CL. Formation of t-Bid is required for the CL dependent oligomerization of Bax and Bak. Cytochrome *c* is detached from oxidized CL and released into the cytosol. OM, outer membrane; IM, inner membrane; IMS, intermembrane space.

Elongation of the isolation membrane is mediated by two ubiquitin-like protein-conjugation systems. Conjugation of Atg12 to Atg5 allows the formation of the Atg12-Atg5-Atg16 complex, which itself functions as an E3 ligase for LC3. LC3 is the mammalian ortholog of Atg8 and is conjugated to phosphatidylethanolamine (PE) in a reaction involving Atg7 (E1-like) and Atg3 (E2-like) to form membrane-bound LC3-II. Lipidated LC3-II is recruited to both the outer and inner surfaces of the autophagosomal membrane and remains on the autophagosomes until fusion with lysosomes. LC3-II contains basic surface patches which bind to CL (Figure 5A; Chu et al., 2013). Consequently, reducing CL levels by knockdown of genes involved in CL biosynthesis causes reduced mitophagy in primary neurons, SH-SY5Y, and HeLa cells (Chu et al., 2013). Recent data suggest that also LC3B, a second member of the Atg8 family, is also specifically recruited to the OM by its interaction with externalized CL (Antón et al., 2016).

### CL peroxidation triggers cytochrome *c* release during apoptosis

Cytochrome *c*, mediates electron transfer from complex III to complex IV in the respiratory chain. A second function has been discovered during apoptosis, where cytochrome *c* acts as a catalyst for peroxidation of cardiolipin (CL). Externalization of CL on the IMS side of the IM increases availability of CL for cytochrome *c*. It has been proposed that conformational changes alter the interaction of CL with cytochrome *c* causing a change in the coordination of heme (Rajagopal et al., 2012; Vincelli et al., 2013). The resulting increase in peroxidase activity targets the closely bound unsaturated fatty acids in CL and results in a substantial increase in oxidized CL (Abe et al., 2011). Cytochrome *c* loses its interaction with oxidized CL, allowing cytochrome *c* release during apoptosis (Figure 5B; He, 2010). CL peroxidation precedes the internal/mitochondrial apoptotic pathway (see below) and peroxidized CL was also found to be a major contributor to the opening of the Mitochondrial Permeability Transition Pore (MPTP). The MPTP consists of the OM protein VDAC, the IM protein adenine nucleotide transporter (ANT) and the peripherally associated IMS protein peptidyl-prolyl cis-trans isomerase cyclophilin D. Direct interactions of CL with ANT explain the role of peroxidized CL in triggering the opening of the MPTP (Schlame et al., 2012; Powers et al., 2013).

A major contributor of cytochrome *c* peroxidase activity has been identified in the protein p66^Shc^. p66^Shc^ is a splice variant of the growth factor adapter Shc (Francia et al., 2009). Activation of the p66^Shc^ pathway was found in hypergylcemia, which significantly increases the severity of cardiovascular diseases. Activated PKCβ induces phosphorylation of p66^Shc^ (Pinton et al., 2007). Phosphorylated p66^Shc^ translocates from the cytosol into the mitochondria where it binds to components of the TOM and TIM protein translocases. Proapoptotic stimuli destabilize this complex and induce the release of p66^Shc^. During apoptosis, p66^Shc^ plays an active role in supporting the peroxidase activity of cytochrome *c* and the generation of reactive oxygen species. Therefore, p66^Shc^ might have an essential role in the oxidation of CL.

### Role of CL during execution of apoptosis

Apoptosis is a controlled process of cell death and maintains the homeostasis of cell populations in tissues and occurs as a defense mechanism in immune reactions or in damaged cells. A role for apoptosis in the heart is described during cardiac development, in ischemia, infarction and in the end stage of heart failure (Bennett, 2002). Apoptosis is executed on two main apoptotic pathways: the extrinsic and the intrinsic pathway. The extrinsic pathway is triggered by the activation of the death receptor such as CD95/FAS/APO-1 in the plasma membrane, followed by the formation of the death-inducing signaling complex (DISC) and the subsequent activation of caspase-8. Caspase-8-mediated processing of Bid to form tBid is necessary to trigger the intrinsic pathway. The intrinsic apoptotic pathway is triggered, when diverse stimuli converge at the mitochondria and induce Bcl-2 family proteins. Bcl-2 proteins are involved in the permeabilization of the outer membrane and the release of pro-apoptotic factors, including cytochrome *c* and SMAC/DIABLO. It has been speculated that the externalization of CL into the outer membrane forms a binding platform for the recruitment of multi-protein complexes, which are required for the execution of apoptosis.

Proteins of the Bcl-2 family play a key role in the regulation of apoptosis and can be divided into the pro-apoptotic proteins (Bax and Bak), the anti-apoptotic (Bcl-2, Bcl-X_L_, Bcl-W, and Mcl1) and the pro-apoptotic BH3-only proteins (Bid, Bim, Bad, Puma and Noxa). The balanced homeostasis of Bcl-2 family proteins is an important regulator of apoptosis. CL has been suggested to serve as a receptor to recruit tBid to the mitochondrial outer membrane (Figure 5B). tBid binding to the mitochondria was significantly decreased in mitochondria from cells deficient in CL (Lutter et al., 2000). Upon membrane binding, tBid promotes Bax membrane insertion. The recruitment and oligomerization of Bax and Bak in the outer mitochondrial membrane was also found to be a CL dependent process (Sorice et al., 2004; Lovell et al., 2008; Lucken-Ardjomande et al., 2008). CL-mediated changes in the structure and curvature of the membrane may be a prerequisite for Bax and Bak oligomerization and pore formation.

Execution of apoptosis depends on caspases, a family of cysteine proteases, which are synthesized in the cell as inactive procaspases. In an amplifying proteolytic cascade, active caspases cleave, and activate, other procaspases, resulting in the targeted degradation of key proteins in the cell. Procaspase-8 predominantly localizes in the cytosol. Apoptotic triggers induce the translocation of active caspase-8 to the mitochondria. Mitochondrial recruitment was shown to be dependent on the presence of CL on the outer mitochondrial membrane (Figure 5B). Lymphoblastoid cells from BTHS patients have been shown to be resistant to apoptotic triggers (Gonzalvez et al., 2008).

### Role of CL in inflammasome activation

Myocardial injury is commonly associated with inflammatory signaling. Upregulation of cytokines (TNF), interleukins (IL-1, IL-6), and chemokines (MCP-1) has been observed in heart failure. Inflammatory signaling was shown to increase fibrosis, is involved in myocardial remodeling, myocyte hypertrophy and decreased contractility and has been considered as a driving force for heart failure progression. The inflammasome is a cytosolic protein complex consisting of the receptor NLRP3, the procaspase-1 and ASC, and is essential for caspase-1 activation (Butts et al., 2015). Upon inflammasome activation, caspase-1 is activated and triggers the processing of pro-IL18 and pro-IL-1β into their active forms (Gurung et al., 2015). In cardiac myocytes, IL-1β induces calcium release from the sarcoplasmic reticulum, causing cardiac deficient contractility. The caspase-1-mediated inflammatory form of a programmed cell death (pyroptosis) is responsible for loss of cardiomyocytes and causes heart failure progression. Knockout of NLRP3 in mice results in smaller infarct sizes in an experimental model of acute myocardial infarction (Mezzaroma et al., 2011).

The inflammasome is activated by external molecular signals summarized as PAMPs (pathogen associated molecular patterns, LPS, proteoglycans, double stranded RNA) or endogenous signals, which are called DAMPS (danger associated molecular patterns, ATP, DNA, Chaperones). The finding of mitochondrial derived DAMPs (mito-DAMPs) including ROS, NAD^+^ and mitochondrial Ca^2+^ release lead to the hypothesis that mitochondrial dysfunction is a contributor to inflammasome activation (Guo et al., 2015; Chakraborty et al., 2017). Mitochondria play a prominent role for inflammasome activity as they provide CL, which was found to bind to the leucin rich LRR domain in NLRP3. In an *in vitro* setting, CL was found sufficient for activation of NLRP3. In CLS1 knockdown cells with reduced CL levels, NLRP3 activity was significantly alleviated (Iyer et al., 2013).

## Conclusions

The mitochondrial phospholipid CL is essential for a large array of functions. Therefore, CL forms multiple interactions with different proteins. As the interaction of CL with proteins serves multiple different functions, a uniform binding site has not been identified. In many of the described binding sites, CL's phosphate groups forms electrostatic interactions with positively charged residues, particularly with Arginine or Lysine (Rytömaa and Kinnunen, 1995). The acyl chains form hydrophobic interactions with Leucin, Isoleucine, and Valine (Kalanxhi and Wallace, 2007). A recent analysis of 62 CL interacting proteins also revealed a role for Glycine in the binding sites, indicating an increased requirement for structural flexibility in the CL binding site (Planas-Iglesias et al., 2015).

CL is a structural constituent of the respiratory chain and required for efficient respiration. A defect in the CL-biogenesis as found in BTHS patients, causes a structural remodeling of the respiratory supercomplexes and a reduction in respiratory performance. Reduced activity of the succinate dehydrogenase affects not only respiration but also the TCA cycle, which is a central hub for the entire cellular metabolism. Mitochondria are integrated in cellular signaling pathways, which monitor mitochondrial function and trigger an adequate nuclear response. Increased generation of ROS caused by the structural changes in the respiratory chain has been described in many models of BTHS. Persistent exposure to ROS is potentially harmful for the cell. Therefore, the cell closely monitors ROS levels in order to trigger defense mechanisms. ROS is an integrative part of a large number of cellular signaling pathways, and affects a wide variety of biological processes including response to hypoxia, apoptosis, autophagy, cell proliferation and differentiation. Several signaling pathways are dependent on CL directly. PKC family proteins are involved in maintaining cardiac structure and function. Some members of this family locate to the mitochondria and require CL for activation. If deficient PKC signaling contributes to BTHS pathology needs to be investigated. CL externalization is evident in mitophagy and apoptosis. Upon stress signals CL forms a binding platform for signaling molecules like LC3, which promotes the engulfment of defective mitochondria into autophagosomes and for pro-apoptotic molecules like caspse-8, Bax, Bak, and tBid. Defective mitophagy and apoptosis has been documented in cellular models for BTHS, but the physiological impact for BTHS patients remains obscure. Most interestingly, the CL molecule itself may even serve as a signaling molecule. A large number of different fatty acids can be bound to four positions within the CL molecule, giving rise to a highly diversified CL pool. The specific function of CL species is vastly unexplored. Interesting new data have elucidated that CL itself serves as a substrate for the generation of lipid mediators. Correlating these new mediators with specific signaling function will be the start of an expanding research field.

## Author contributions

The author confirms being the sole contributor of this work and approved it for publication.

### Conflict of interest statement

The author declares that the research was conducted in the absence of any commercial or financial relationships that could be construed as a potential conflict of interest.
